# SHAP combined with machine learning to predict mortality risk in maintenance hemodialysis patients: a retrospective study

**DOI:** 10.3389/fmed.2025.1615950

**Published:** 2025-07-07

**Authors:** Peng Shu, Xia Wang, Zhuping Wen, Jie Chen, Fang Xu

**Affiliations:** The Central Hospital of Wuhan, Tongji Medical College, Huazhong University of Science and Technology, Wuhan, Hubei, China

**Keywords:** hemodialysis, predictive modeling, machine learning, mortality risk, SHAP

## Abstract

**Background:**

Patients undergoing maintenance hemodialysis face a high mortality rate, yet effective tools for predicting mortality risk in this population are lacking. This study aims to develop an interpretable machine learning model to predict mortality risk among maintenance hemodialysis patients.

**Methods:**

A retrospective analysis was conducted on clinical data from 512 maintenance hemodialysis patients treated at The Central Hospital of Wuhan between January 2021 and October 2024. The dataset included 50 feature variables. The data were split into a training set (70%) and a test set (30%). Five machine learning models—Random Forest, Extreme Gradient Boosting, Support Vector Machine, Logistic Regression, and K-Nearest Neighbor—were trained and evaluated for predicting patient mortality risk, using metrics such as the F1 score, precision, accuracy, AUC-ROC, and recall. SHAP values were used to assess the contribution of each feature in the best-performing model.

**Results:**

The K-Nearest Neighbor model achieved the highest AUC-ROC of 0.9792 (95% CI: 0.9600–0.9929). SHAP analysis identified key factors influencing predictions, including dialysis duration, creatinine levels, white blood cell ratio, blood phosphorus concentration, and unconjugated iron.

**Conclusion:**

The K-Nearest Neighbor model demonstrated high efficacy in predicting mortality risk among hemodialysis patients. SHAP analysis highlighted critical risk factors. While these findings show promise for future clinical research, they should be interpreted with caution due to the study’s retrospective design and the need for external validation.

## 1 Introduction

Chronic kidney disease (CKD) is a non-communicable disease predominantly attributed to diabetes and hypertension (1). As of 2017, the global prevalence of CKD was reported to be 9.1%, with a corresponding mortality rate of 4.6% (2). Within the Chinese population, the prevalence of CKD is estimated at 8.2% (3), with a mortality rate of 6.95%, which exceeds that of the global population (4). Notably, patients undergoing end-stage renal replacement therapy exhibit a significantly elevated risk of mortality compared to the general population, with approximately 20% of dialysis patients succumbing each year (5). Various high-risk factors contribute to mortality among individuals on maintenance hemodialysis (MHD). The early identification of these risk factors, coupled with appropriate care and treatment interventions, is essential for enhancing the quality of life and extending the survival of affected patients (6). In the realm of healthcare, artificial intelligence (AI) and machine learning, distinguished by their capabilities in data management and processing, present novel opportunities for advancing healthcare delivery and care models (7). Machine learning algorithms possess the capability to process extensive volumes of clinical data, identify potential risk factors, and construct predictive models that assist physicians in more accurately assessing patients’ conditions (8). Within the domain of vascular diseases, machine learning models have been effectively employed for risk assessment in areas such as coronary artery disease, peripheral vascular disease, and renal disease, yielding favorable outcomes (9–11). Nevertheless, traditional machine learning models frequently suffer from a lack of interpretability, a challenge that constrains their application in clinical practice (12).

To address this limitation, the Local Interpretable Model-agnostic Explanations (LIME) and SHAP (SHapley Additive exPlanations) values have been introduced to elucidate the predictions of machine learning models. LIME primarily provides local explanations, focusing on the interpretability of individual samples and relying on perturbation sampling. SHAP values, derived from the Shapley values in game theory, are able to quantify the contribution of each feature to the model’s predictions, thereby providing interpretable prediction results. SHAP offers both global and local explanations, with a more rigorous theoretical foundation, but at a relatively higher computational complexity (13). The objective of this study was to develop and validate a predictive model based on machine learning, utilizing SHAP values to interpret outcomes and predict mortality risk in hemodialysis patients. The model’s capability to quantify the contribution of each feature to its predictions enhances the transparency of machine learning models by elucidating their “black box” nature. The SHAP methodology not only identifies the most influential features in forecasting mortality risk among hemodialysis patients but also elucidates the interactions and combined effects of these features on model predictions.

In this investigation, five machine learning models were constructed to predict mortality risk in hemodialysis patients, from which the optimal model was selected and subsequently interpreted using the SHAP approach. The K-nearest neighbor model (KNN) was determined to be the most suitable model. This algorithm represents a straightforward and intuitive supervised learning approach that is extensively employed in both classification and regression tasks. Its fundamental principle posits that within the feature space, the class or value of a given sample can be inferred from the classes or values of its K nearest neighboring samples. KNN algorithms have found considerable application within the medical domain (14).

The study aims to furnish clinicians with an advanced decision-support tool to facilitate the early identification of patients at elevated risk of mortality, thereby enabling timely interventions to improve patient prognosis and ultimately extend patient lifespan.

## 2 Materials and methods

### 2.1 Study design

This research employed a retrospective cohort study design, wherein demographic and biochemical data from hemodialysis patients at the Hemodialysis Center of The Central Hospital of Wuhan were collected retrospectively, spanning the period from January 1, 2021 to October 30, 2024. Data collection was conducted using a purposive sampling method. Patients were classified into two groups—death and survival—based on their mortality status. The study received ethical approval from the Ethics Committee of The Central Hospital of Wuhan, under approval number WHZXKYL2024-115.

Follow-Up: Patients receiving hemodialysis at our hospital from January 1, 2021, to October 31, 2024, were included. Follow-up began on each patient’s first recorded hemodialysis date and ended on October 31, 2024, or at the time of death, whichever occurred first. In this study, the follow-up duration for patients ranged from 3 to 221 months.

Patient censoring: Patients were censored from the study upon transferring to another healthcare facility for dialysis or becoming lost to follow-up, thereby precluding the determination of their survival outcomes.

### 2.2 Inclusion and exclusion criteria

(1) Inclusion Criteria: The study included participants who met the following criteria: (1) aged 18 years or older; (2) undergoing a minimum of two dialysis sessions per week; and 3) receiving dialysis sessions with a duration of at least 3 h each.

(2) Exclusion Criteria: Participants were excluded from the study if they met any of the following conditions: (1) receiving dialysis through a temporary catheter; (2) having more than 30% of clinical information missing; or (3) having left the institution without access to patient survival outcomes.

### 2.3 Feature selection

Patient demographics were counted based on medical records in our electronic data case system, involving a total of 69 characteristic variables. A series of clinical indicators and laboratory findings were collected to comprehensively assess the health status of hemodialysis patients. These included basic information (Age, gender, survival outcome, frequency of dialysis, etiologic diagnosis, education, type of vascular access, hypertension, diabetes mellitus, secondary hyperparathyroidism, hyperphosphatemia, heart failure, gout, cerebral infarction, myocardial infarction, and age at dialysis), Physiologic indices include body mass index and various blood cell counts, while biochemical markers cover a range of lipids, vitamins, iron levels, hormones, proteins, enzymes, and kidney function indicators.

### 2.4 Data processing

In this study, we conducted missing value processing and multiple imputation on data collected from hemodialysis patients. It is important to note that the missing data in our dataset are likely to be Missing Not At Random (MNAR), as the absence of certain laboratory tests or clinical assessments may be related to the patients’ health status or other factors that influenced their decision to undergo these tests. For instance, patients with less severe conditions might have opted out of certain tests, or economic constraints might have prevented some patients from completing all recommended assessments. To address the missing data, we initially excluded columns exhibiting more than 50% missing data to mitigate the potential impact of high missing rates on the analysis. Ultimately, 50 feature variables were retained.

Subsequently, we performed multiple imputation using the miceforest library in Python, generating 10 complete datasets through five iterations. This approach was chosen to comprehensively account for the uncertainty associated with missing values, especially given the potential non-random nature of the missingness. A random seed of 42 was established to ensure the reproducibility of the results. The robustness of the imputed datasets was assessed through model training and evaluation metrics, including the Normalized Root Mean Square Error (NRMSE) for regression tasks and the Receiver Operating Characteristic Area Under the Curve (AUC-ROC) for classification tasks. The datasets demonstrating optimal performance were selected for further analysis. Subsequently, the robustness of the data was assessed; for each continuous variable, the first quartile (Q1) and the third quartile (Q3) were calculated, along with the interquartile range (IQR = Q3 − Q1). Outlier boundaries were established, with values falling below Q1 − 1.5 × IQR or exceeding Q3 + 1.5 × IQR classified as outliers. These outliers were addressed by replacing values below the lower boundary with Q1 and values above the upper boundary with Q3. Finally, multiple imputation was performed utilizing the miceforest library in Python, generating 10 complete datasets through five iterations to comprehensively account for the uncertainty associated with missing values.

In the imputation process, a random seed of 42 was established to guarantee the reproducibility of the results. Subsequently, valid datasets were assessed through model training and evaluation metrics, specifically the Normalized Root Mean Square Error (NRMSE) for regression tasks and the Receiver Operating Characteristic Area Under the Curve (AUC-ROC) for classification tasks. The datasets demonstrating optimal performance were selected for further analysis; specifically, the dataset exhibiting the lowest NRMSE was chosen for regression problems, while the dataset with the highest AUC-ROC was selected for classification problems, thereby ensuring the accuracy and reliability of the model.

### 2.5 Constructing machine learning models

The dataset was randomly partitioned into training and test sets with a 7:3 ratio. Logistic Regression (LR), Random Forest (RF), K-Nearest Neighbors (KNN), Support Vector Machine (SVM), and Extreme Gradient Boosting (XGBoost) models were developed using Python software (version 3.13.2). The training and validation datasets were imported, and clinical demographics along with laboratory test results from the training set were utilized as predictors to construct the models, with patient mortality serving as the target variable.

### 2.6 Data standardization

To ensure the stability and effectiveness of model training, we standardized the continuous variables in the dataset.

Specifically, we used the StandardScaler module in Python (version 3.13.2), which is based on the Z-score standardization method. This method transforms each continuous variable into a distribution with a mean of 0 and a standard deviation of 1. This process helps to eliminate differences in scale and numerical range among different features, thereby preventing certain features from dominating the model training process due to their larger numerical ranges. The data standardization process was performed as follows: (1) The dataset was split into training and test sets. (2) The Standard Scaler was applied to the continuous variables in the training set to compute the mean and standard deviation. (3) The training set was standardized using these computed statistics. (4) The test set was standardized using the same statistics derived from the training set to ensure consistency. Handling Data ImbalanceData imbalance was addressed using oversampling techniques. Specifically, the Synthetic Minority Over-sampling Technique (SMOTE) was applied to the training set to balance the class distribution. This step was performed after standardization to ensure that the synthetic samples generated were consistent with the standardized data distribution. Model training and validation optimal parameters for each model were identified through grid search and five-fold cross-validation. Upon finalizing the models, validation was conducted using the validation dataset, and performance metrics such as the area under the receiver operating characteristic curve (AUC), sensitivity, specificity, accuracy, recall, and F1 score were computed for each model.

### 2.7 Feature interpretation

To interpret and rank the features of the training models, the SHAP package was employed to assess the contribution of each feature to the model. Following the selection of the best-performing model, SHAP values were further utilized to visualize and analyze the significance of the features.

### 2.8 Statistical analysis

Python (version 3.13.2) was employed for data processing and statistical analysis. Categorical variables were represented as frequencies and percentages and were compared using either Fisher’s exact test or the chi-square test. For continuous variables, the Shapiro-Wilk test was initially applied to assess normality. If the data conformed to a normal distribution, comparisons were made using the independent samples *t*-test, with results expressed as mean ± standard deviation. For data not conforming to a normal distribution, comparisons were made using non-parametric methods, and results were reported as median with interquartile range (first and third quartiles). A *p*-value of less than 0.05 was considered indicative of statistical significance.

## 3 Results

### 3.1 Comparison of patients’ general information

**FIGURE 1:**
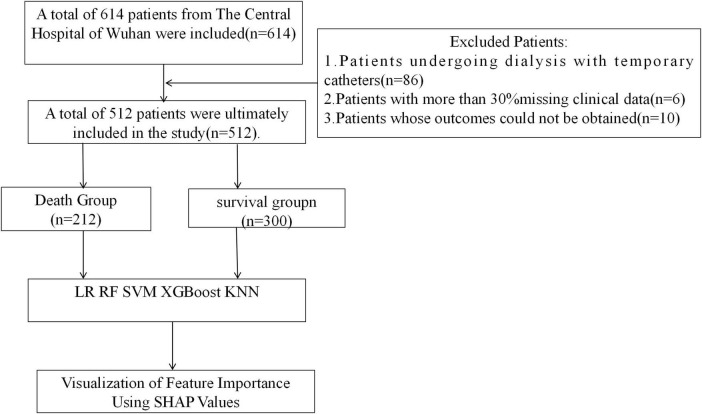
Flowchart of study design.

A total of 614 patients were enrolled in this study and finally 512 patients were enrolled in the study, 300 in the survivor group and 212 in the mortality group (Figure 1). 50 characteristics were included in the study [Age, gender, education level (EL), vascular access type (VAT), hypertension (HTN), diabetes mellitus (DM), secondary hyperparathyroidism (SHPT), hyperphosphatemia (HP), heart failure (HF), gout (Gout), cerebral infarction (CI), myocardial infarction (MI), dialysis frequency (DF), dialysis age (DV), and other characteristics], [heart failure (HF), gout (Gout), cerebral infarction (CI), myocardial infarction (MI), dialysis frequency (DF), dialysis age (DV)], physiologic indices [body mass index (BMI), white blood cell count (WBC), red blood cell count (RBC), Hematocrit (HCT), Monocyte count (MONO), platelet count (PLT), Lymphocyte Count (LYMPH), Neutrophil Count (NEUT), Hemoglobin Concentration (HGB), Potassium (K), Sodium (Na), Chlorine (Serum Cl), Calcium (Ca), Phosphorus (P)] as well as biochemical markers [Total Iron Binding Capacity (TIBC), Serum Iron (SI), Unbound Iron (UI), Parathyroid Hormone (iPTH), Glucose (GLU)], urea (URE), creatinine (CRE), uric acid (UA), total carbon dioxide (TCO2), alpha hydroxybutyrate dehydrogenase (αHBDH), lactate dehydrogenase (LDH), creatine kinase (CK), creatine kinase isoenzyme (CKMB), total bilirubin (TBIL), direct bilirubin (DBIL), indirect bilirubin (IBIL), alanine aminotransferase (ALT), aspartate aminotransferase (AST), gamma-glutamate aminotransferase (GGT), total protein (TP), albumin (ALB), globulin (GLB), and albumin-globulin ratio (AGR)].There was a significant difference in educational qualifications, type of pathway, diabetes mellitus, blood phosphorus, and creatinine between the 2 groups (*p* < 0.05) (Table 1).

**TABLE 1:** Comparison of the characteristics of the two groups of patients.

	Survivor group (*n* = 300)	Death group (*n* = 212)	Statistical value	*P*
Gender			2.260	0.133
Female	108 (36.0%)	62 (29.2%)		
Male	192 (64.0%)	150 (70.8%)		
EL			22.794	0.000*
Illiteracy	30 (10.0%)	44 (20.8%)		
Primary education	52 (17.3%)	38 (17.9%)		
Junior high school	96 (32.0%)	40 (18.9%)		
High school/secondary school	54 (18.0%)	38 (17.9%)		
Three-year college	22 (7.3%)	8 (3.8%)		
Undergraduate and above	46 (15.3%)	44 (20.8%)		
Vascular access			17.061	0.000*
Tunneled cuffed catheter	44 (14.7%)	64 (30.2%)		
Arteriovenous fistula	256 (85.3%)	148 (69.8%)		
Hypertension			1.537	0.215
No	28 (9.3%)	28 (13.2%)		
Yes	272 (90.7%)	184 (86.8%)		
Diabetes			4.043	0.044*
No	216 (72.0%)	134 (63.2%)		
Yes	84 (28.0%)	78 (36.8%)	5.33	0.021*
Secondary hyperparathyroidism				
No	206 (68.7%)	166 (78.3%)		
Yes	94 (31.3%)	46 (21.7%)		
Hyperphosphatemia			12.444	0.000*
No	210 (70.0%)	178 (84.0%)		
Yes	90 (30.0%)	34 (16.0%)		
Heart failure			0.948	0.33
No	206 (68.7%)	136 (64.2%)		
Yes	94 (31.3%)	76 (35.8%)		
Gout				
No	290 (96.7%)	202 (95.3%)	0.319	0.572
Yes	10 (3.3%)	10 (4.7%)	0.319	0.572
Cerebral infarction			0.018	0.893
No	246 (82.0%)	172 (81.1%)		
Yes	54 (18.0%)	40 (18.9%)		
Myocardial infarction			27.744	0.000*
No	292 (97.3%)	178 (84.0%)		
Yes	8 (2.7%)	34 (16.0%)		
Number of dialysis (times/week)			3.291	0.07
2	68 (22.7%)	64 (30.2%)		
3	232 (77.3%)	148 (69.8%)		
Age (years)	58.0854 ± 13.2568	62.1601 ± 12.3315	7.812	0.000*
BMI (kg/m^2^)	22.586 ± 3.657	22.160 ± 2.985	1.445	0.149
WBC (10^∧^9/L)	6.784 ± 2.356	7.254 ± 3.220	−1.809	0.071
RBC (10^∧^12/L)	3.064 ± 0.798	3.090 ± 0.633	−0.413	0.679
HCT (L/L)	27.739 ± 6.437	27.645 ± 5.377	0.179	0.858
MONO (10^∧^9/L)	0.470 ± 0.251	0.488 ± 0.259	−0.778	0.437
PLT (10^∧^9/L)	188.154 ± 77.618	187.597 ± 68.586	0.086	0.932
LYMP (10^∧^9/L)	1.280 ± 0.921	1.140 ± 0.491	2.213	0.027*
NEUT (10^∧^9/L)	4.839 ± 2.087	5.414 ± 3.053	−2.378	0.018*
HGB (g/L)	90.618 ± 23.658	88.750 ± 17.601	1.024	0.306
K (mmol/L)	4.946 ± 0.832	4.941 ± 0.863	0.066	0.947
Na (mmol/L)	139.790 ± 3.916	139.639 ± 3.346	0.469	0.64
Serum Cl (mmol/L)	102.562 ± 5.813	103.096 ± 5.050	−1.106	0.269
Ca (mmol/L)	2.235 ± 0.252	2.231 ± 0.258	0.178	0.858
p (mmol/L)	1.787 ± 0.507	1.680 ± 0.582	2.157	0.032*
TIBC (μmol/L)	44.707 ± 8.276	44.782 ± 8.585	−0.098	0.922
SI (μmol/L)	10.261 ± 4.970	9.854 ± 4.312	0.987	0.324
UI (μmol/L)	34.291 ± 8.504	34.746 ± 8.194	−0.609	0.543
PTH (pg/mL)	60.219 ± 93.885	39.987 ± 34.739	3.416	0.001*
GLU (g/L)	6.471 ± 2.948	7.116 ± 3.269	−2.288	0.023*
URE (mmol/L)	24.209 ± 21.544	21.499 ± 8.482	1.973	0.049*
CRE (μmol/L)	794.262 ± 338.309	682.964 ± 307.572	3.868	0.000*
UA (μmol/L)	396.397 ± 121.743	373.807 ± 117.092	2.115	0.035*
TCO2 (mmol/L)	21.712 ± 3.962	22.476 ± 4.206	−2.073	0.039*
αHBDH (U/L)	172.657 ± 42.427	176.217 ± 55.973	−0.781	0.435
LDH (U/L)	235.443 ± 81.830	244.806 ± 161.400	−0.777	0.438
CK (U/L)	234.099 ± 320.536	126.474 ± 383.221	3.345	0.001*
CKMB (U/L)	10.636 ± 6.580	10.181 ± 5.687	0.835	0.404
TBIL (μmol/L)	7.301 ± 10.054	9.814 ± 30.809	−1.145	0.253
DBIL (μmol/L)	2.486 ± 6.547	4.086 ± 21.755	−1.038	0.300
IBIL (μmol/L)	4.675 ± 3.863	5.715 ± 9.265	−1.543	0.124
ALT (U/L)	15.020 ± 12.995	16.537 ± 26.971	−0.759	0.448
AST (U/L)	18.210 ± 9.761	19.461 ± 16.410	−0.993	0.322
GGT (U/L)	27.989 ± 18.324	40.558 ± 115.028	−1.577	0.116
TP (g/L)	68.062 ± 9.852	68.534 ± 10.795	−0.505	0.614
ALB (g/L)	37.718 ± 6.337	37.085 ± 6.640	1.083	0.279
GLB (g/L)	30.141 ± 5.546	31.173 ± 5.963	−1.986	0.048*
AGR	1.288 ± 0.261	1.209 ± 0.219	3.675	0.000*

*p < 0.05.

### 3.2 Comparison between the performance of different models

**FIGURE 2:**
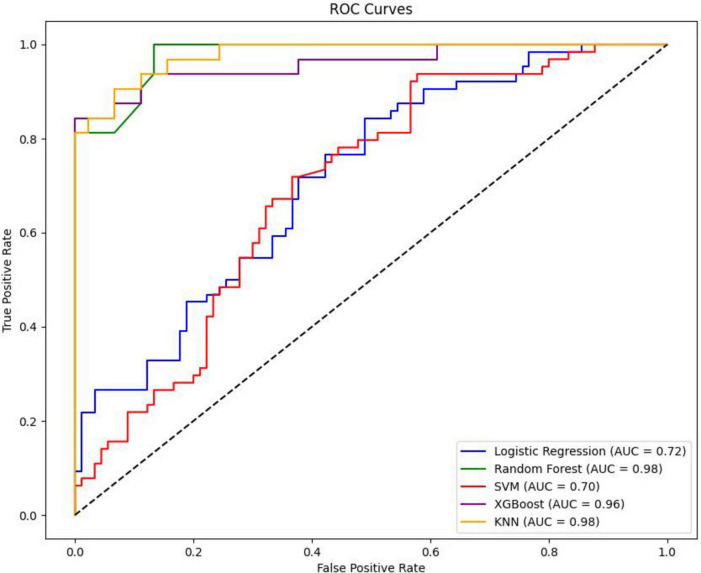
Plot of AUC comparison between different models.

In this study, five distinct machine learning models were developed. Among these, the K-Nearest Neighbors model demonstrated superior performance, achieving an Area Under the Curve of 0.9792 (95% Confidence Interval: 0.9600–0.9929), as presented in Table 2. The optimal parameters for each model are detailed in Table A1. The model exhibiting the highest AUC value was designated as the best-performing model in this study, as illustrated in Figure 2. The sensitivity analysis of the best model is provided in Table A2.

**TABLE 2:** Comparison of indicators between different models.

Mould	AUC	F1 score	Accuracy	Accurate	Recall rate
LR	0.7163	0.6389	0.6623	0.5750	0.7188
	(0.6300–0.7923)	(0.5496–0.7222)	(0.5907–0.7338)	(0.4675–0.6829)	(0.6111–0.8209)
RF	0.9771	0.8710	0.8961	0.9000	0.8438
	(0.9572–0.9910)	(0.8000–0.9280)	(0.8442–0.9416)	(0.8135–0.9667)	(0.7535–0.9299)
SVM	0.6984	0.6309	0.6429	0.5529	0.7344
	(0.6174–0.7799)	(0.5372–0.7180)	(0.5649–0.7208)	(0.4444–0.6623)	(0.6207–0.8358)
XGBoost	0.9601	0.8710	0.8961	0.9000	0.8438
	(0.9255–0.9897)	(0.8036–0.9231)	(0.8505–0.9416)	(0.8225–0.9655)	(0.7500–0.9310)
KNN	0.9792	0.8955	0.9091	0.8571	0.9375
	(0.9600–0.9929)	(0.8376–0.9449)	(0.8636–0.9545)	(0.7678–0.9385)	(0.8689–0.9855)

### 3.3 Feature importance interpretation in KNN models

**FIGURE 3:**
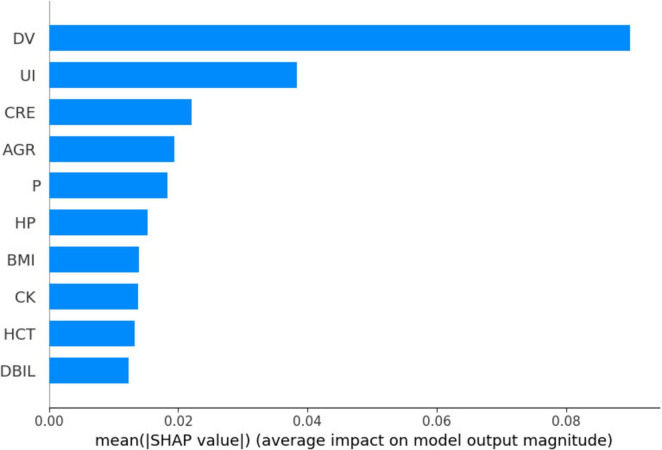
Importance ranking of mortality risk in the KNN model.

**FIGURE 4:**
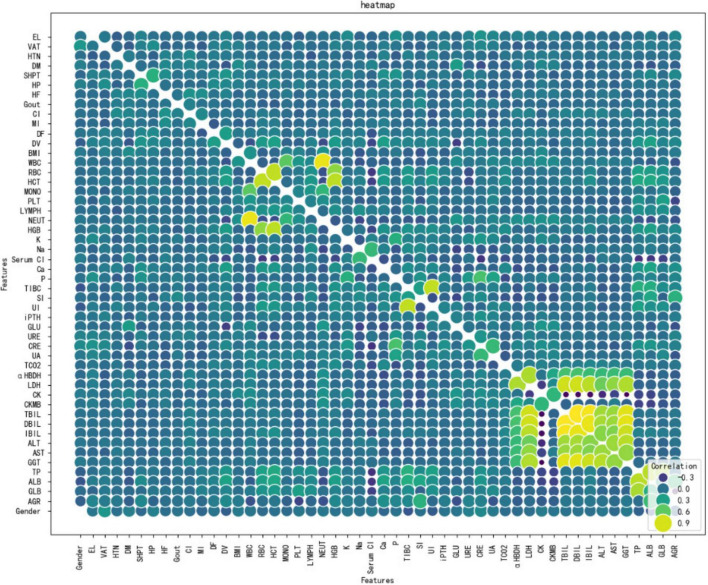
Bubble heat map of correlations between significance features.

Figure 3 shows Dialysis age (DV), unconjugated (UI), creatinine (CRE),Albumin/Globulin Ratio (AGR), and blood phosphorus concentration (P) are important characteristics for the risk of death in MHD. In order to better show the relationship between the variables, this study used bubble heat map to show the relationship between the characteristics (Figure 4).

**FIGURE 5:**
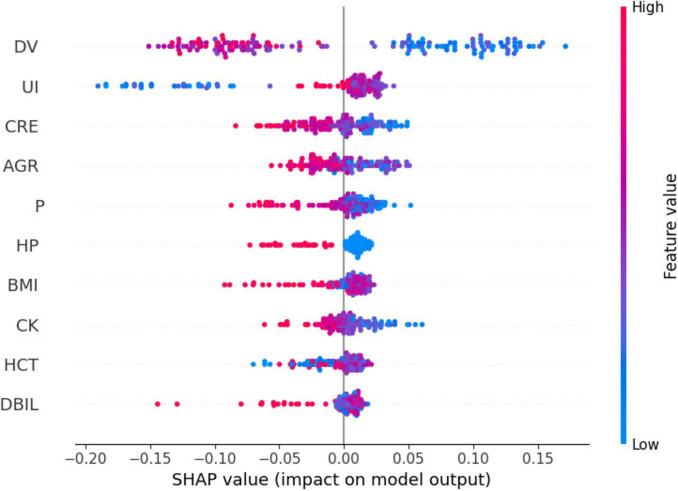
Scatter plot of SHAP values for different features.

Figure 5 presents a summary plot of the characteristic SHAP values. For dialysis age (DV), it was observed that elevated values (indicated in red) generally contributed to an increase in the model output, whereas lower values (indicated in blue) were associated with a decrease in the model output. In contrast, for unbound iron (UI), the impact of high and low values on the model output was more variable. However, on average, higher values exhibited a slight positive effect.

**FIGURE 6:**
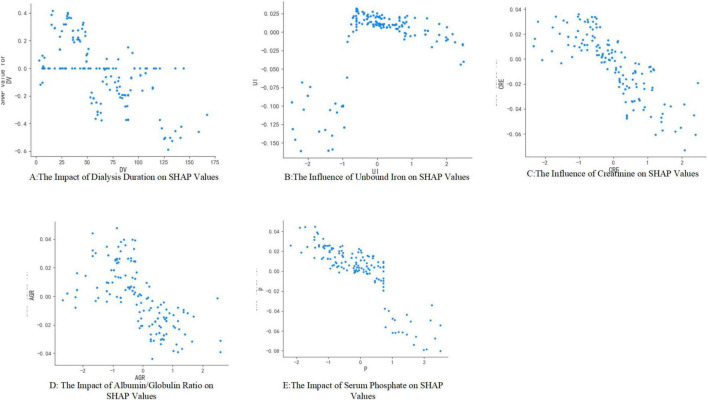
SHAP dependency graph for different features.

Figure 6 illustrates the impact of individual characteristics on the model. Specifically, dialysis age (DV), creatinine (CRE), albumin-globulin ratio (AGR), and blood phosphorus (P) exhibit a negative influence on the model. In contrast, unconjugated iron (UI) demonstrates a positive effect.

**FIGURE 7:**
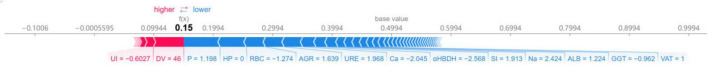
Single patient predictive SHAP force plot.

We employed K-Nearest Neighbors models to visualize individual patient mortality risk predictions. Specifically, the arrows illustrate the influence of each factor on the prediction outcomes. Features that elevate the risk of brain death are depicted in red, whereas those that mitigate the risk are shown in blue. The length of each stripe represents the significance of the corresponding feature in the prediction process; a longer stripe indicates a greater contribution of that feature to the prediction. By aggregating the effects of each factor, we calculated the respective prediction score for each feature. Figure 7 presents the contribution value of each feature to the accurate prediction of mortality risk in hemodialysis patients. For instance, in the case of the first patient, the predicted risk of mortality was 15%.

## 4 Discussion

According to the American Society of Kidney Diseases, the mortality rate for maintenance hemodialysis patients in the United States is 16.7% (15), whereas in Europe it is 13.1% (16). A study conducted in China reports a crude mortality rate of 8.8% among hemodialysis patients (17). It is imperative to implement appropriate measures to mitigate the risk of mortality in this patient population. Our study identified several factors associated with mortality risk in maintenance hemodialysis patients, including dialysis duration, levels of unconjugated iron, creatinine, white blood cell count, and blood phosphorus concentration. Notably, the relationship between dialysis duration and mortality risk exhibited a U-shaped curve: patients undergoing dialysis for less than 1 year and those with a duration of 5–10 years experienced higher mortality peaks, whereas those with 1–5 years of dialysis demonstrated a relatively stable mortality rate (18, 19).

This outcome may be attributed to the combined effects of both dialysis duration and patient age. Newly initiated dialysis patients frequently present with severe malnutrition, anemia (hemoglobin levels below 90 g/L), electrolyte imbalances such as hyperkalemia, failure to achieve dry weight, and psychological conditions including anxiety and depression (20). Furthermore, there is a markedly elevated risk of early cardiovascular events, such as acute heart failure, and infections, including catheter-associated sepsis (18). As the duration of dialysis increases, patients are more likely to experience disturbances in calcium and phosphorus metabolism and muscular dystrophy, which may elevate the mortality risk among long-term dialysis patients. Cardiovascular events remain the leading cause of mortality, potentially linked to complications arising from prolonged dialysis (21). The relationship between age at the initiation of dialysis and mortality risk in patients undergoing maintenance hemodialysis is complex and dynamic. In the early stages, risks are primarily associated with maladaptation and acute complications, whereas in the mid- to long-term, metabolic disorders and chronic pathologies predominate. Consequently, healthcare professionals can mitigate all-cause mortality in hemodialysis patients through a series of staged interventions, including nutritional support, cardiovascular management, and tumor screening, alongside personalized strategies such as optimization of the primary disease and careful selection of vascular access. Furthermore, precision in dialysis management can be enhanced by integrating biomarkers with emerging technologies.

In studies of iron metabolism, unbound iron refers to free iron ions not sequestered by transferrin. The presence of unbound iron, which can result from either iron deficiency or iron overload, is closely linked to increased mortality risk in patients undergoing maintenance hemodialysis through mechanisms involving cardiovascular injury, oxidative stress, and inflammatory responses. Iron deficiency, in particular, is associated with coronary artery calcification, potentially due to accelerated vascular calcification arising from reduced activity of iron-dependent enzymes such as anti-calcitonin (22). Conversely, iron overload leads to a significant increase in unconjugated iron, which is highly oxidative and directly contributes to lipid peroxidation, endothelial damage, and tissue iron deposition, thereby elevating the incidence of cardiovascular events under oxidative stress conditions (23).

Creatinine serves as a fundamental indicator of nutritional and muscular status in patients undergoing maintenance hemodialysis Both persistently low levels and a dynamic decline in creatinine are associated with an increased risk of mortality. In dialysis patients, renal function is compromised, rendering creatinine clearance almost entirely reliant on dialysis. Consequently, blood creatinine levels predominantly reflect muscle mass. A significant association exists between low blood creatinine levels and muscle atrophy, as well as protein-energy wasting. Studies have demonstrated that patients who succumbed exhibited lower blood creatinine levels compared to survivors, with a 12.6-fold increase in mortality risk for each 1 mg/dL decrease in creatinine (hazard ratio [HR] = 12.60, 95% confidence interval [CI] 0.66–241.35) (24). Elevated creatinine levels (e.g., > 10 mg/dL) are indicative of better muscle mass and nutritional status, correlating with more favorable clinical outcomes (25). Furthermore, patients experiencing a decline of more than 15% in serum creatinine over a 2-year period have a 5-year mortality rate of 40.3%. To maintain creatinine within the optimal range (6–10 mg/dL) and enhance patient prognosis, comprehensive clinical interventions, including nutritional supplementation, anti-inflammatory therapy, and dialysis optimization, are essential (26). Although serum creatinine holds significant predictive value, its concentration is predominantly influenced by the glomerular filtration rate (GFR), a vital marker of renal function. In clinical practice, serum creatinine is extensively utilized to estimate GFR due to its convenient measurement and cost-effectiveness. Nevertheless, it is crucial to acknowledge that serum creatinine levels may be affected by factors beyond GFR, including muscle mass, dietary intake, and specific medications, potentially leading to inaccuracies in GFR estimation (27). Consequently, integrating multiple clinical indicators and employing machine learning models is essential for achieving a more comprehensive assessment of patient prognosis.

In recent years, the albumin-globulin ratio (AGR) has emerged as a valuable prognostic marker for assessing nutritional status and chronic inflammation in patients undergoing maintenance hemodialysis. A study involving 320 MHD patients revealed that the 5-year all-cause mortality rate was significantly elevated in the low AGR group (< 1.21) compared to the high AGR group (32.98% vs. 10.3%). Even after adjusting for age and comorbidities, a low AGR was associated with a 2.74-fold increased risk of all-cause mortality (hazard ratio [HR] = 2.740, 95% confidence interval [CI] 1.08–6.64) (28). The cardiovascular effects of the albumin-globulin ratio may be mediated through inflammatory pathways. Specifically, a low globulin level results in decreased colloid osmotic pressure and tissue edema, which can exacerbate cardiac insufficiency. Additionally, it diminishes the reserve of antioxidants, such as thiol groups, thereby increasing oxidative stress. Conversely, elevated globulin levels indicate the activation of pro-inflammatory cytokines, including interleukin-6 (IL-6) and tumor necrosis factor-alpha (TNF-α), which may facilitate the progression of microinflammation to systemic inflammation, thereby accelerating atherosclerosis and protein-energy wasting. AGR is a significant predictor of mortality risk in patients undergoing maintenance hemodialysis and is valued for its ability to integrate the dual pathophysiological processes of nutrition and inflammation. Consequently, it is recommended that an AGR of ≥ 1.2 be established as a management target through dynamic monitoring, stratified intervention, and multidisciplinary management, including nutritional fortification, anti-inflammatory therapy, and dialysis optimization. Furthermore, it is advised that serum albumin, globulin, and inflammatory markers such as C-reactive protein and IL-6 be monitored every 3 months to allow for the dynamic adjustment of nutritional support in MHD patients.

The relationship between blood phosphorus levels and mortality in patients undergoing maintenance hemodialysis is characterized by a complex time-dose-effect dynamic. This relationship exhibits a U-shaped risk at absolute phosphorus levels and an independent hazard with dynamic variability. Numerous studies have demonstrated an association between blood phosphorus levels and mortality in MHD patients. Mortality rates are lowest when blood phosphorus levels are maintained within the range of 3.5–5.5 mg/dL (1.13–1.78 mmol/L). However, all-cause mortality significantly increases when blood phosphorus levels exceed 5.0 mg/dL (1.6 mmol/L), with a marked escalation in risk observed at levels above 7.0 mg/dL (2.26 mmol/L), where the hazard ratio (HR) reaches 2.02 (29). A blood phosphorus level of less than 3.5 mg/dL (1.13 mmol/L) is associated with malnutrition, and patients with such levels exhibit a 5-year mortality rate of 32.98%, alongside a 2.74-fold increase in corrected risk (30). Mechanistically, elevated phosphorus levels inhibit endothelial nitric oxide synthase and elevate markers of oxidative stress, contributing to increased arterial stiffness. Additionally, high phosphorus levels upregulate pro-inflammatory cytokines such as interleukin-6 (IL-6) and tumor necrosis factor-alpha (TNF-α), further elevating mortality risk when C-reactive protein levels exceed 5 mg/L. Phosphorus accumulation also facilitates the production of uremic toxins, such as indolephenol sulfate, which synergistically interact with fibroblast growth factor 23 (FGF-23) to impair myocardial mitochondrial function. This impairment subsequently leads to left ventricular hypertrophy, as indicated by a left ventricular mass index (LVMI) greater than 130 g/m^2^ (29, 31). Serum phosphorus levels play a critical role in the mortality of patients undergoing maintenance hemodialysis primarily through pathways involving vascular calcification, the inflammation-dystrophy axis, and metabolic toxicity. Clinically, a stratified management approach is essential. For patients exhibiting hyperphosphatemia, intensive dialysis coupled with pharmacological interventions is recommended. Conversely, for those with hypophosphatemia, the focus should be on addressing malnutrition and enhancing long-term outcomes by dynamically monitoring the coefficient of variation in serum phosphorus levels.

Currently, the prediction of survival rates for patients with end-stage renal disease predominantly depends on indices such as the Davies Index, Khan Index, and the Charlson Comorbidity Index (CCI). In addition, other comorbidity indices like the Index of Coexistent Diseases (ICED) and the Index of Chronic Disease Diagnoses (ICDD) are also utilized. However, these indices exhibit certain limitations: they encompass numerous variables, rendering them less suitable for clinical application and statistical analysis. The Davies Index suffers from ambiguous definitions of comorbidities, resulting in problematic weight allocation. The Khan Index assigns equal weights to each comorbidity, which may not accurately reflect their relative impact. Furthermore, the CCI tends to overestimate the weight of certain comorbidities (32). The interpretable machine learning model that we have developed can be integrated into clinical record systems for practical use in clinical settings. The model predicts the risk of death for individual patients by analyzing their data (Figure 7).

The findings of our study highlight certain features that significantly contribute to the optimal model; however, these may not align with some clinical research outcomes. Beyond the features identified in our study, it is crucial to consider additional factors in maintenance hemodialysis patients. These factors include blood pressure, fluid status, dialysis dose (e.g., Kt/V), anemia, bone mineral parameters, inflammation markers, and residual kidney function, which have not been explicitly incorporated into our model but warrant careful consideration.

Previous research has established that the nutritional status of hemodialysis patients is a critical determinant of patient mortality. Specifically, a decrease in serum albumin levels is significantly associated with an elevated risk of death within 6 months (6). In our study, no significant difference in serum albumin levels was observed between the deceased and survivor groups. However, variations were noted in the albumin/globulin ratio and globulin levels between these groups, potentially attributable to the administration of albumin during dialysis. Consequently, it is imperative to closely monitor and address the nutritional status of these patients.

Several studies have demonstrated that dialysis adequacy constitutes a significant risk factor influencing mortality among hemodialysis patients. High-dose dialysis, defined as a Kt/V greater than 1.4, has been associated with a reduction in all-cause mortality in patients undergoing maintenance hemodialysis. This effect is particularly pronounced in subgroups of patients younger than 65 years or those with a dialysis duration exceeding 60 months, where the risk of cardiovascular disease (CVD) also shows a marked decrease (33, 34).

Similarly, blood pressure significantly influences the survival rates of hemodialysis patients. Empirical evidence indicates a reduction in the incidence of cardiovascular events among patients with systolic blood pressure (SBP) levels of 101–110 mmHg (HR 0.647, 95% CI 0.455–0.920), 111–120 mmHg (HR 0.663, 95% CI 0.492–0.894), 121–130 mmHg (HR 0.747, 95% CI 0.569–0.981), and 131–140 mmHg (HR 0.757, 95% CI 0.596–0.962) (35). Our study did not incorporate blood pressure indicators. Consequently, it is imperative to consider variations in a patient’s blood pressure when formulating clinical decisions. This approach is essential to ensure the provision of comprehensive preventive measures for patients.

### 4.1 Limitations of the study

Several limitations of our study should be acknowledged when interpreting the results. First, this study was conducted as a single-center, retrospective analysis and did not include external validation. This design may limit the generalizability and external validity of the findings, as single-center studies may not adequately reflect the characteristics of patient populations in different regions or healthcare settings. Future research should consider conducting multicenter, large-sample, prospective cohort studies to enhance the accuracy and generalizability of the models.

Second, the data in our study are likely subject to Missing Not At Random (MNAR) issues. The absence of certain laboratory tests or clinical assessments may be related to the patients’ health status or other factors that influenced their decision to undergo these tests. This non-random missingness could introduce bias, as the missing data may systematically differ from the observed data. For example, patients with milder conditions or those with financial constraints may be more likely to have missing data. Although we employed multiple imputation techniques to address missing data, these methods rely on assumptions about the missing data mechanism. Given the potential MNAR nature of our missing data, it is possible that our imputation model did not fully capture the underlying patterns of missingness. This could affect the accuracy and generalizability of our findings. Future research should consider more advanced methods for handling MNAR data, such as pattern mixture models or selection models, which can explicitly account for the non-random nature of missing data. Additionally, sensitivity analyses could be conducted to assess the robustness of the results under different assumptions about the missing data mechanism.

Furthermore, our study may have been affected by unmeasured confounding variables. These include nominal variables (such as gender and race) and treatment-related factors (such as treatment protocols and medication use),which may have influenced the study outcomes. Although we controlled for known confounders in our analysis, unmeasured confounders could still introduce bias into the interpretation of the results. Future research should aim to reduce the impact of these potential confounders through more comprehensive data collection and analysis methods.

While our study provides some insights into the relevant issues, its findings need to be further validated in broader research contexts. Future studies should employ multicenter, large-sample, prospective cohort designs, as well as more advanced statistical methods, to improve the accuracy and reliability of the results. The objective of this modeling is to support clinical decision-making; however, the final treatment decision remains the responsibility of the attending nephrologist. This tool is not designed to supplant clinical judgment.

## 5 Conclusion

We developed and evaluated five distinct machine learning models to predict mortality risk among hemodialysis patients, identifying the K-nearest neighbor algorithm as the most effective. To elucidate the factors influencing mortality risk, we employed SHAP values for interpretability. Our analysis revealed that mortality risk is associated with variables such as dialysis vintage, white blood cell ratio, creatinine levels, blood phosphorus concentration, and serum iron. The integration of the KNN algorithm with SHAP values offers a transparent and interpretable framework for risk prediction, holding significant potential for application in future clinical research. This approach aids clinicians in implementing timely interventions and provides comprehensive insights for the long-term management of hemodialysis patients, ultimately contributing to the reduction of mortality risk. However, despite the model’s high predictive efficacy in the single-center data, its clinical value should still be interpreted with caution. The inherent limitations of observational studies mean that the risk factors identified by the model need to be combined with clinical judgment and cannot replace the comprehensive assessment of individual patients by physicians.

## Data Availability

The raw data supporting the conclusions of this article will be made available by the authors, without undue reservation.

## Appendix

**TABLE A1:** Optimal model parameters.

**Model**	**Best Parameters**
Logistic regression	‘{‘C’: 1, ‘solver’: ‘liblinear’}’
Random forest	‘{‘max_depth’: 20, ‘min_samples_split’: 2, ‘n_estimators’: 200}’
SVM	‘{‘C’: 0.1, ‘kernel’: ‘linear’}’
XGBoost	‘{‘learning_rate’: 0.1, ‘max_depth’: 7, ‘n_estimators’: 100}’
KNN	‘{‘n_neighbors’: 10, ‘weights’: ‘distance’}’

**TABLE A2:** Comparison of various indicators in subgroup analysis.

Featur	Subgrou	Sample size	Accuracy	Recall	Precision	F1	AUC
Gender	1	103	0.8835	0.9167	0.8462	0.8800	0.9742
Gender	0	51	0.9608	1.0000	0.8889	0.9412	0.9929
VAT	1	120	0.9000	0.9070	0.8298	0.8667	0.9807
VAT	0	34	0.9412	1.0000	0.9130	0.9545	0.9560
HTN	1	134	0.8955	0.9231	0.8276	0.8727	0.9737
HTN	0	20	1.0000	1.0000	1.0000	1.0000	1.0000
DM	1	47	0.9574	0.9231	1.0000	0.9600	0.9853
DM	0	107	0.8879	0.9474	0.7826	0.8571	0.9817
SHPT	0	116	0.8966	0.9600	0.8276	0.8889	0.9794
SHPT	1	38	0.9474	0.8571	1.0000	0.9231	0.9643
HP	0	126	0.9048	0.9643	0.8438	0.9000	0.9806
HP	1	28	0.9286	0.7500	1.0000	0.8571	0.9750
HF	0	105	0.8857	0.9000	0.8182	0.8571	0.9723
HF	1	49	0.9592	1.0000	0.9231	0.9600	1.0000
Gout	0	147	0.9048	0.9322	0.8462	0.8871	0.9777
Gout	1	7	1.0000	1.0000	1.0000	1.0000	1.0000
CI	0	122	0.9016	0.9245	0.8596	0.8909	0.9792
CI	1	32	0.9375	1.0000	0.8462	0.9167	0.9654
MI	1	14	0.8571	1.0000	0.8182	0.9000	0.9111
MI	0	140	0.9143	0.9273	0.8644	0.8947	0.9812
DF	2	44	0.8636	1.0000	0.7500	0.8571	0.9658
DF	3	110	0.9273	0.9130	0.9130	0.9130	0.9851
